# The larva of *Oecetis tripunctata* (Fabricius, 1793) (Trichoptera, Leptoceridae)

**DOI:** 10.3897/zookeys.445.8153

**Published:** 2014-10-13

**Authors:** Johann Waringer, Wolfram Graf

**Affiliations:** 1 Department of Limnology and Oceanography, Faculty of Life Sciences, University of Vienna; 2 Institute of Hydrobiology and Aquatic Ecology Management, University of Natural Resources and Applied Life Sciences, Vienna, Austria

**Keywords:** Description, distribution, larva, identification, West Paleartic fauna

## Abstract

*Oecetis tripunctata* is a widely distributed leptocerid in Europe, ranging from the Iberian and Apennine peninsulas and the Central and Western European highlands to the plains of Eastern Europe. The long, single-bladed mandibles are indicative for a predacious lifestyle. This paper describes the previously unknown larva of *Oecetis tripunctata*. Information on the morphology of the 5th larval instar is given, and the most important diagnostic features are illustrated. A synoptic key for the European species of *Oecetis* is also provided. In the context of existing identification keys the larva of *Oecetis tripunctata* keys together with *Oecetis intima* and *Oecetis notata*. *Oecetis tripunctata* is separated from the other two species by the fact that a double row of long setal fringes is lacking at the hind tibiae and that several long setae are present on the protrochantinus.

## Introduction

From Europe, ten species of genus *Oecetis* McLachlan, 1877 are known (Malicky 2004, 2005). However, the larvae of only seven species were described up to now: *Oecetis furva* (Rambur), *Oecetis intima* McLachlan, *Oecetis lacustris* (Pictet), *Oecetis notata* (Rambur), *Oecetis ochracea* (Curtis), *Oecetis struckii* Klapálek (unvalid synonym = *Paroecetis* Lestage) and *Oecetis testacea* (Curtis). References for the larval descriptions available are listed in Table 1. The larvae of *Oecetis canariensis* Brauer, *Oecetis grazalemae* Gonzalez & Iglesias and *Oecetis tripunctata* (Fabricius) were unknown. Recently, however, W. Graf managed to collect larvae of *Oecetis tripunctata* at the Thaya river in the northern part of Lower Austria. *Oecetis tripunctata* is a widely distributed leptocerid in Europe, ranging from the British Isles and the Iberian and the Apennine peninsulas and the Central and Western European highlands to the plains of Eastern Europe (Graf et al. 2008). Across Asia, this species ranges even to Bali, with many records in the countries between. *Oecetis tripuncatata* is probably the caddisfly species with the widest distribution range currently known worldwide (Malicky 2005, 2009). This taxon was described by Fabricius (1793) in the genus *Phryganea*, redescribed as *Setodes punctatella* (Rambur, 1842), as *Oecetis buitenzorgensis* (Ulmer, 1951) and as *Oecetis alexanderi* (Kumanski, 1976). *Oecetis buitenzorgensis* was declared as synonym of *Oecetis tripunctata* (Fabricius, 1793) by Malicky (2009b) whereas *Oecetis alexanderi* was declared as synonym of *Oecetis tripunctata* by Kumanski (1988) (Yang and Morse 2000; Morse 2014). With our description of its larva, proposed here, the identification of eight out of ten European *Oecetis* species is now possible even without an adult male as frequently practiced within caddisflies studies.

**Table 1.** Synoptic key for the currently known European *Oecetis* (incl. *Paroecetis*) larvae (5th instars).

Species/character	Mandible with 2 cutting edges (Fig. 13)?	Mandible sickle-shaped, 1 cutting edge (Fig. 3)?	Head with distinct dark patches (Fig. 14)?	Hind tibiae with 2 long setal fringes (Fig. 16)?	Number of long setae on protrochantin (Figs 6, 15)	Basal seta of 2nd and 3rd tarsal claw rudimentary? (Figs 9, 17)	References
*Oecetis furva*^2^	no	yes	yes	no	1	no	Wallace et al. 2003, Waringer and Graf 2011
*Oecetis intima*	no	yes	no^1^	no	1	yes	Lepneva 1964
*Oecetis lacustris*	no	yes	yes	no	several	yes	Wallace et al. 2003, Waringer and Graf 2011
*Oecetis notata*	no	yes	no	yes	several	no	
*Oecetis ochracea*^2^	no	yes	yes	no	1	yes	
*Oecetis struckii*	yes	no	yes	no	1	no	Wiberg-Larsen and Waringer 1998
*Oecetis testacea*	no	yes	yes	yes	1	no	Wallace et al. 2003, Waringer and Graf 2011
*Oecetis tripunctata*	no	yes	no	no	several	yes	present paper

^1^ Larvae in brackish water; very pale head pattern may be present in some larvae (Lepneva 1964). Southeastern species (Pontic province, eastern plains, Caucasus, Caspic depression, Asia Minor; Graf et al. 2008).

^2^ In *Oecetis ochracea* 1–4 setae are present at each side of the mesoventer; such setae are lacking in *Oecetis furva*.

## Material and methods

The larvae were sampled on 18 December 2012 by Wolfram Graf in the Thaya river at Hohenau, a short distance upstream of its confluence with the March river in Lower Austria. The catchment of the Thaya is situated within the granite and gneiss complex of the Bohemian highlands. Due to its low slope, the river meanders strongly and has created some scenic, deeply carved valleys descending up to 150 m steeply downwards from the figau of the surrounding highlands. The watershed area of the Thaya is 13.319 km² with an average discharge of 43.9 m³ s^–1^ and a Strahler stream order of seven. Some river stretches of the Thaya situated within the Czech Republic have been transformed in reservoirs used for irrigation, drinking water supply and hydroelectric power plants (Waringer 2003). A hand net was used to collect larvae of *Oecetis tripunctata* in the Thaya river at Hohenau in the northern part of the federal state of Lower Austria on 18 December 2012 (48°36'04"N, 16°56'10"E, 161 m above sea level). The material was preserved in 70% ethanol. A Nikon Labophot 2 microscope and a Nikon SMZ 1500 binocular microscope with DS-Fi1 camera and NIS-elements D 3.1 image stacking software for combining 8–42 frames in one focused image were used to study and photograph the larvae.

Species association was enabled by the fact that all other five *Oecetis* species reported from Austria (Malicky 2009a: *Oecetis furva*, *Oecetis lacustris*, *Oecetis notata*, *Oecetis ochracea*, *Oecetis testacea*) are well known in the larval stage (e.g., Wallace et al. 2003; Waringer and Graf 2011), and the new taxon is morphologically very different from the other species. Additionally, close to our larval collecting site more than 300 adults of *Oecetis tripunctata* were sampled by mobile light traps.

The 5th instar larvae of *Oecetis tripunctata* are deposited in the collection of J. Waringer (Vienna, Austria) and in the collection of W. Graf (Vienna, Austria). Comparative material of other *Oecetis* species included the following 5th instar larvae: *Oecetis furva* (n = 5), *Oecetis lacustris* (n = 2), *Oecetis notata* (n = 6), *Oecetis ochracea* (n = 2), *Oecetis struckii* (n = 3) and *Oecetis testacea* (n = 3) (all taxa from the collection of J. Waringer, Vienna, Austria). We used the morphological terminology by Waringer and Graf (2011) and Wallace et al. (2003).

## Results

### Oecetis tripunctata

Taxon classificationAnimaliaTrichopteraLeptoceridae

(Fabricius, 1793)

 Description of the 5th instar larva

##### Diagnosis.

Mandible sickle-shaped, with only one cutting edge; head capsule without distinct dark patches; hind tibiae without double row of long setal fringes; number of long setae on protrochantinus is > 1; basal setae on 2nd and 3rd tarsal claw rudimentary.

##### Biometry.

Body length of 5th instar larvae ranging from 2.7 to 4.5 mm, head width from 0.66 to 0.70 mm (n = 15).

##### Head.

**Figures 1–6.**
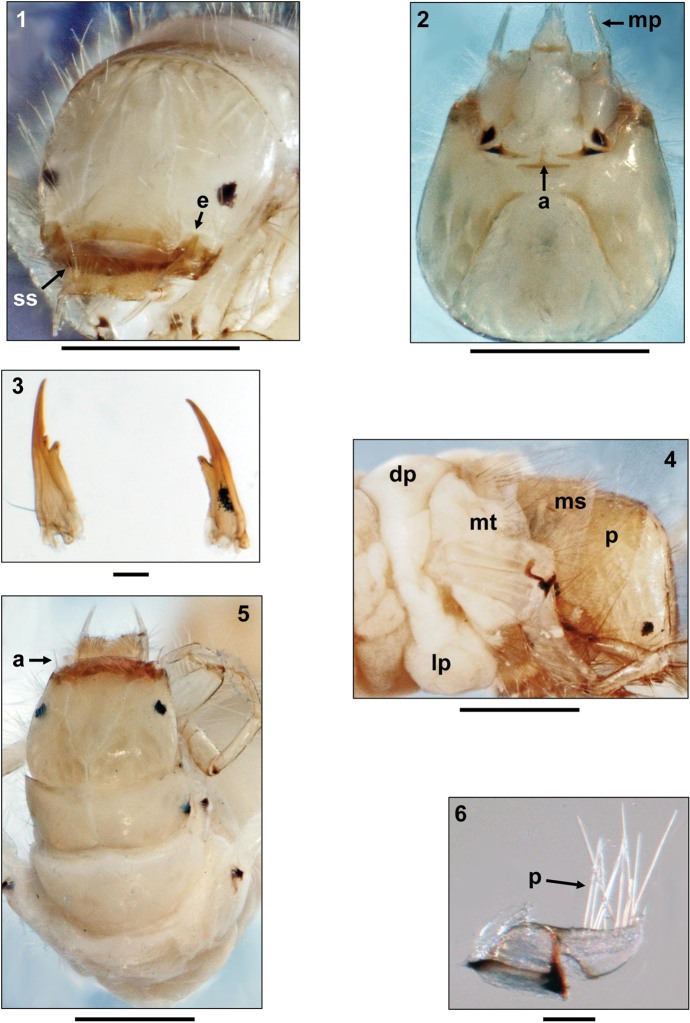
*Oecetis tripunctata* (Fabricius, 1793), 5th instar larva. **1** Head, dorsal view (e = subocular ecdysial line bending dorsally and meeting frontoclypeal suture; ss = secondary setae on labrum) **2** Head, ventral view (a = ventral apotome; mp = maxillary palp) **3** Mandibles, dorsal view **4** Head, thorax and 1st abdominal segment, right lateral view (p = pronotum; ms = mesonotum; mt = metanotum; dp = dorsal protuberance; lp = lateral protuberance) **5** Head and thorax, dorsal view (a = antenna) **6** Right protrochantin, lateral view (p = pale setae). Scale bars: 0.5 mm (except Figs **3**, **6:** 0.1 mm).

Head capsule surface very smooth, roundish and hypognathous with pale yellow coloration. Light muscle attachment spots on frontoclypeus and parietalia very indistinct (Figs 1–2). White ring present around eyes (Fig. 1). In addition to complete set of primary setae, head capsule densely covered by pale secondary setae, especially at anterolateral corners, dorsally of eyes and over frontoclypeus (Figs 1, 4, 5). Frontoclypeus elongated, narrow, with very shallow central constriction at eye level (Figs 1, 5). Subocular ecdysial line running from foramen occipitale to lateral section of parietalia, ventrally to the eyes. Anteriorly of the eyes the subocular ecdysial line bends dorsally, eventually meeting frontoclypeal suture (Fig. 1e). Antennae slender, approximately six times longer than its width at widest section, situated at extreme anterior end of parietalia and originating from a socket-like ridge (Fig. 5a). Labrum light brown, quadrangular, with anterior median notch (Fig. 1); in addition to 6 pairs of primary setae, with numerous secondary setae on dorsal surface (Fig. 1ss). Maxillary palps very long, distinctly protruding labrum (Fig. 2mp). Ventral apotome trapezoidal in shape (Fig. 2a), pale yellow with light brown anterior border; apotome not separating parietalia posteriorly. Mandibles single-bladed, sickle-shaped, with only 1 cutting edge; with sharp terminal tooth and 1–2 subapical secondary teeth (Fig. 3).

##### Thorax.

Pronotum covering posterior section of head, light yellowish-brown, semitransparent, without distinct markings and muscle attachment spots (Fig. 5); dense continuous row of straight, pale setae along anterior border; pronotal surface densely covered by high number of pale setae (Figs 1, 4). Pleural sclerites pale, semicircular, with thin, blackish-brown ventral margins; anteriorly, with pale, large, ear-like protrochantin bearing numerous pale setae (Fig. 6p). Prosternal horn absent.

Mesonotum completely covered by two sclerites, yellowish and paler than pronotum, with distinct markings and muscle attachment spots (Figs 4, 5); dense cover of pale setae on the surface and along anterior border. Pleural sclerites pale, with thin, blackish-brown ventral margin (Fig. 4). Mesoventer without setae.

Metanotum without sclerotization except by pleural sclerites and with dense dorsal setal cover; pleural sclerites arrangement as on mesonotum (Fig. 4).

**Figures 7–12.**
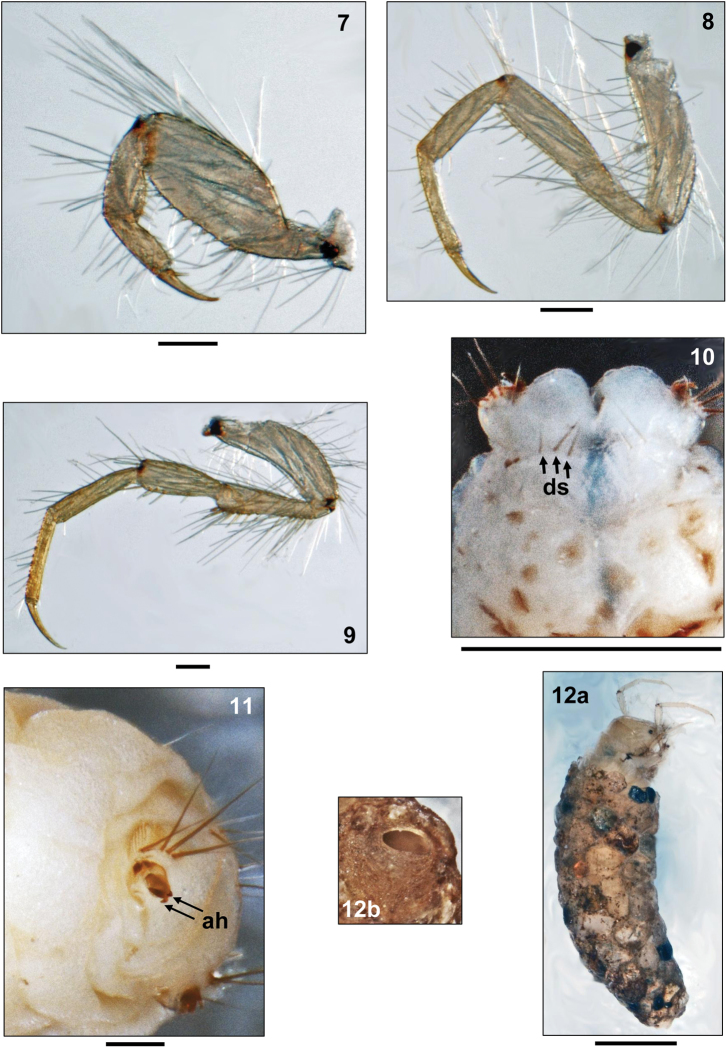
*Oecetis tripunctata* (Fabricius, 1793), 5th instar larva. **7** Left 1st leg, posterior view **8** Left 2nd leg, posterior view **9** Left 3rd leg, posterior view **10** Tip of abdomen, dorsal view (ds = dorsal setae) **11** Tip of abdomen, left posterolateral view (ah = 2 accessory hooks on anal claw) **12a** Larva and case, right lateral view **12b** Tip of larval case, posterior view. Scale bars: 0.1 mm (except Fig. **10:** 0.5 mm and Fig. **11:** 1 mm).

Legs yellowish, with very numerous setae, especially on coxae, trochanters and femora (Figs 7–9); tibiae and tarsi undivided and without central constrictions. Femur of foreleg much wider than those of mid- and hind legs. Claw of mid leg curved and not hook-shaped (Fig. 8). Long setal fringes for swimming lacking on hind legs; distal section of hind trochanter broadened and club-like (Fig. 9).

##### Abdomen.

First abdominal segment with one dorsal (Fig. 4dp) and two lateral protuberances (Fig. 4lp), the latter with very pale and inconspicuous lateral sclerites. Lateral fringe present from segments 3–7, consisting of extremely short, pale hairs. Dorsum of 9th abdominal segment with 6–8 setae (Fig. 10ds). Anal prolegs medium brown, anal claws dark brown, each with two small dorsal accessory hooks (Fig. 11ah). Anal region without rows of spines and tooth-edges figs (Fig. 11).

Gills single-filamented; dorsal gills present at most from 2nd segment (presegmental position) to 3rd segment (presegmental position); ventral gills only at 3rd segment (presegmental); lateral gills absent.

##### Case.

Larval case 3.0–3.7 mm long (n = 15), curved, tapered (width at anterior opening 1.2–1.5 mm and at posterior opening 0.6–0.7 mm), consisting of mix of mineral particles of unequal grain size (Fig. 12a). Posterior case opening partly closed by terminal silken membrane with oval foramen 0.2 mm wide and arranged transversally; ventral lip of membrane slightly protruding, creating an upward-directed twist of the foramen (Fig. 12b).

###### Synoptic key for the currently known European *Oecetis* larvae (final instars; Table 1)

Within genus *Oecetis*, *Oecetis tripunctata* keys together with *Oecetis intima* McLachlan, 1877 and *Oecetis notata* (Rambur, 1842) (Table 1). *Oecetis tripunctata* is easily separated from the other two species by the fact that a double row of long setal fringes is lacking at the hind tibiae (Fig. 9) and that several long setae are present on the protrochantinus (Fig. 6).

###### Biological remarks

Our collection time of the larvae is in accordance with the reported spring to summer emergence and flight periods of the species; the emergence period is short and mostly limited to two months or less (Graf et al. 2008). In a light trap study from the nearby river March, we observed *Oecetis tripunctata* to be on the wing only from June 27th to August 3rd (Waringer and Graf 2006).

As pointed out by Wiggins (1996), *Oecetis* larvae are bottom-dwellers covering a wide range of habitats from lentic to lotic environments and may be even collected from brackish waters (e.g., *Oecetis intima*; Lepneva 1964). In Europe, the preferred habitats are lowland rivers with low current velocities, e.g. the Raab and the March systems in Austria.

**Figures 13–17.**
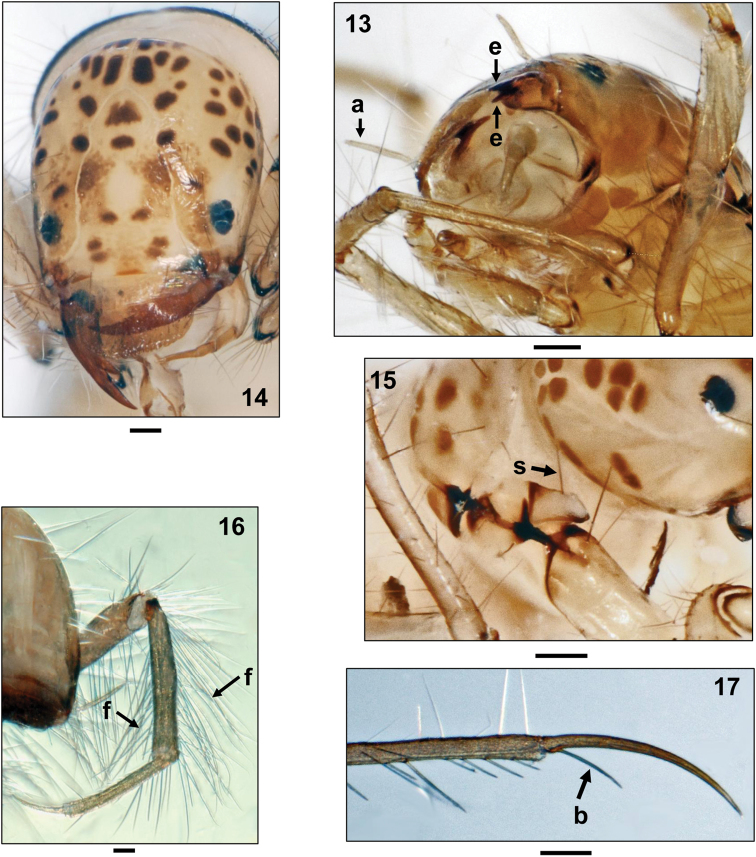
**13** *Oecetis struckii* Klapálek, 1903, 5th instar larva, left ventrolateral view (a = antenna, e = two cutting edges of the mandible) **14** *Oecetis testacea* (Curtis, 1834), 5th instar larva, head, frontal view **15** *Oecetis furva* (Rambur, 1842), 5th instar larva, right propleuron, lateral view (s = long seta on protrochantin) **16** *Oecetis notata* (Rambur, 1842), 5th instar larva, head and 3rd leg, frontolateral view (f = two long setal fringes on hind tibia) **17** *Oecetis furva* (Rambur, 1842), 5th instar larva, distal section of right 3rd tarsus and claw (b = basal seta). Scale bars: 0.1 mm.

The long, single-bladed predatory jaws of *Oecetis tripunctata* and most other known *Oecetis* larvae are unusual among cased caddisflies; they are used for catching worms and chironomid larvae which are ingested whole (Wallace et al. 2003). Interestingly, *Oecetis struckii*, once attributed to genus *Paroecetis* Lestage, is unique in that the mandibles are double-edged (Fig. 13, arrows). Nevertheless, Wiberg-Larsen and Waringer (1998) reported also for this *Oecetis* species only animal remains in the foregut (Testacea, Hydrachnellae, Oribatei, Cladocera, *Asellus*, Chironomidae).

## Supplementary Material

XML Treatment for Oecetis tripunctata
